# Candidate gene and locus analysis of myopia

**Published:** 2007-06-28

**Authors:** Donald O. Mutti, Margaret E. Cooper, Sarah O'Brien, Lisa A. Jones, Mary L. Marazita, Jeffrey C. Murray, Karla Zadnik

**Affiliations:** 1 College of Optometry, The Ohio State University, Columbus, OH; 2 Center for Craniofacial and Dental Genetics, Department of Oral Biology, School of Dental Medicine, University of Pittsburgh, Pittsburgh, PA; 3 University of Iowa, Department of Pediatrics, Iowa City, IA

## Abstract

**Purpose:**

A previous study has reported evidence of a strong linkage, but no association, between *paired box gene 6* (*PAX6*) and myopia. We attempted to replicate these findings and to conduct a candidate gene and locus evaluation of genetic involvement in common forms of myopia.

**Methods:**

Samples were collected from 517 individuals in 123 families with a myopic child participating in the Orinda Longitudinal Study of Myopia or the Contact Lens and Myopia Progression Study. Myopia in the proband children was defined as -0.75 D or more and as being present in both meridians on cycloplegic autorefraction (1% tropicamide). Affected status in parents and siblings was determined by survey. After DNA was extracted from buccal mucosal cells and genotyped using assays for microsatellite markers and single nucleotide polymorphisms (SNPs), DNA was analyzed for linkage disequilibrium. Markers on chromosomes 12 and 18 were selected as regions previously associated with pathological myopia. SNPs were also analyzed in genes where their expression pattern or their association with syndromes conveys myopia as part of the phenotype (*FGF2*, *BDNF*, *COL2A1*, *COL18A1*, and *PAX6*).

**Results:**

The SNP rs1635529 for *COL2A1* on 12q13.11 showed highly significant over-transmission to affected individuals (p=0.00007). No SNP for *FGF2*, *BDNF*, *COL18A1*, or *PAX6* showed significant over-transmission to affected individuals after correction for multiple comparisons. Markers on chromosome 12 and 18 previously associated with pathological myopia also showed no significant associations with the more common form of myopia in this study.

**Conclusions:**

As reported previously by others, *PAX6* showed no association with myopia. Associations in the current analysis are suggestive of involvement of *COL2A1*. Future studies should focus on replication in other samples and in genome-wide approaches.

## Introduction

Myopia is a common, complex trait most often acquired in childhood where the axial length of the eye exceeds its focal length, resulting in reduced distance visual acuity. The costs of providing clear distance vision through spectacles, contact lenses, or refractive surgery are considerable [1]. Myopia has additional public health significance as a risk factor for ocular disease including glaucoma, cataract, and retinal detachment [2-8].

Evidence points to a substantial role for genetic factors in the etiology of myopia. Myopic parents tend to have myopic children [9-12]. Heritabilities calculated from twin studies are high, on the order of 0.8 to nearly 1.0 [13,14]. The role of environment, represented by near visual activity, in the etiology of myopia remains debatable [15], but recent analysis of the contributions of children's near work and the parental history of myopia shows that parental history makes the greater contribution [12]. Near work typically explains little of the variance in refractive error, on the order of 2% to 12% [16-19]. Combinations of environmental and hereditary causes for myopia have also been postulated. Suggestions include a hereditary susceptibility coming from a shared, intensive parental near work environment or that myopic genes may increase susceptibility to the influence of environmental sources of myopia such as near work. Recent studies have either not found evidence for these effects [12] or have not shown a dose-response relationship between increased myopia risk and more near work when comparing children with one, two, or any myopic parents [20].

Yet, differences in the prevalence between groups with supposedly similar genetic makeup but separated geographically and in near work demands suggests that near work may influence refractive error [21]. Researchers in Asia point to their rigorous schooling system and the long hours children spend studying as responsible for the high rates of myopia in Asia, rates that may be on the rise [22-25]. Likewise, the level of education attained, perhaps a marker for near work demand, intellectual aptitude, or both, is a risk factor for myopia [16,26-28]. Interestingly, the higher prevalence rates for myopia in Asia seem consistently related to education [22,23,29,30] but have only been weakly associated with near work itself [30-32]. Unfortunately, a recent report of a significant odds ratio of 1.12 for completing >20.5 h of near work per week in a sample of Singapore 14-15 year-olds is difficult to interpret because of the lack of adjustment for the educational track or aptitude of the students [33]. Therefore, while controversy remains on the relative contributions of heredity and environment to myopia, the literature suggests a substantial contribution from heredity.

In addition to enhancing our understanding of the underlying biology of myopia, a better understanding of genetic factors in myopia might lead to improvements in prediction of the onset, treatment, and, perhaps, prevention. Identification of the genetic factors involved in complex traits is complicated by the involvement of a multiplicity of genes, genetic epistasis, and population heterogeneity. Despite these issues, several research groups have made strides in the last eight years toward identifications of genetic regions of interest with respect to myopia. Typically, the studies have been of families with histories of pathological, more severe degrees of myopia. These regions include 18p11.31 in eight American [34] and 15 Chinese families [35], 12q21-23 in a German/Italian family [36], 17q21-22 in an English/Canadian family [37], 2q37.1 in an American family of Northern European extraction [38], and 7q36 in 21 French and two Algerian families [39]. Linkage for 18p11.31 (marker D18S63) was also found for high myopia in a group of subjects from Sardinia [40]. Evidence for linkage was absent for 18p and weak for 17q and 12q in a subset of the 51 English families with at least two siblings having myopia of -6.00 D or more [41]. Different linked loci may play a role in more common forms of lower levels of myopia. Evidence for linkage was found at 22q12.3 and 1p36 (depending on trait definition) in large samples of Ashkenazi Jewish individuals from New Jersey where affected subjects had at least -1.00 D of myopia in each meridian [42,43]. Regions of interest in high myopia, 18p and 12q, have not appeared to play a strong role in lower levels of myopia [44,45]. When refractive error was analyzed as a continuous trait including hyperopia, evidence for linkage to 11p13 was found in a large sample of British adult twins. However, using single nucleotide polymorphisms (SNPs) covering the *PAX6* gene located in that region, there was no evidence for association with myopia [46]. Therefore, the available evidence suggests heterogeneity for high myopia while leaving open the question of whether there is any overlap between genes that might be responsible for both the rarer forms of high myopia and more common, less severe juvenile onset myopia.

In this study, we examined the role of candidate genes and loci in myopia. We either evaluated loci cited in the above reports from other investigators (except for the most recent) or evaluated based on the possible biological relevance of a candidate gene to myopia. Basic fibroblast growth factor (FGF-2) has been shown to inhibit form deprivation myopia in the chick [47]. Brain-derived neurotrophic factor (BDNF) is a neuroprotective agent involved in neural organization and development during activity-dependent competition [48-51]. Besides its relevance to neural development, its presence in the retina is altered during deprivation [52]. As deprivation is a major paradigm for producing experimental myopia, *brain-derived neurotrophic factor* (*BDNF*) is of interest as a potential genetic marker for myopia. Two clinical conditions have severe myopia in common: Knobloch and Stickler syndromes. Each has been associated with one or more mutations in collagen genes. Among these are *collagen, type XVIII, alpha 1* (*COL18A1*) for Knobloch [53] and *collagen, type II, alpha 1* (*COL2A1*) for Stickler syndrome [54]. Families primarily presenting with high myopia have not shown linkage with the Stickler *COL2A1* locus in several other studies [34,37,39,41].

We used an association approach and a large sample that has been well characterized phenotypically in conjunction with detailed candidate genetic analysis.

## Methods

### Study subjects

Myopic children enrolled in the Orinda Longitudinal Study of Myopia (OLSM) through 2000 [55] and in the Contact Lens and Myopia Progression (CLAMP) Study [56] were eligible for participation. According to the tenets of the Declaration of Helsinki, parents and their children originally supplied informed consents to participate in the respective primary studies then supplied separate consents for collection and analysis of genetic material. Genetic material was obtained using buccal swab kits mailed to the family members. Then from the family, the kits were mailed to the University of Iowa for analysis. Samples were obtained from 517 individuals representing 123 predominantly nuclear pedigrees. The parent-reported ethnic makeup of the sample was as follows: Caucasian (62%), East Asian (13%), Hispanic (8%), African-American (7%), Indian/Pakistani (4%), with the remaining 6% comprised of Native American, Afghan, Filipino, mixed, or other. Of these 123 pedigrees, 23 were trios of parents and proband, 66 had one additional sibling, 26 had two siblings, six had three siblings, and two pedigrees were extended. Of the 517 participants, 342 were affected myopes, 131 were unaffected non-myopes, and the refractive status of 44 was unknown. Of families with concordant affected siblings, 31 had two affected siblings, four had three affected siblings, and two had four affected siblings. There were 62 typed children with no myopia. A previous report on only markers from chromosomes 12 and 18 analyzed a subset of 221 samples from 53 families included in the current analysis [44].

To be classified as affected, probands and siblings in OLSM or CLAMP had to have at least -0.75 D or more myopia in each principal meridian according to the most recent annual measurement of refractive error in the right eye by cycloplegic autorefraction (1% tropicamide). Other non-study siblings and parents were classified as affected according to their responses to survey questions [57].

### DNA extraction and genotyping

DNA was extracted from buccal mucosa cells using described protocols [58]. Additionally, a few swabs were processed using Qiagen's QIAamp Mini Kit protocol (Qiagen, Inc., Valencia CA). For PAGE (polyacrylamide gel electrophoresis) and SSCP (single-stranded conformation polymorphism) assays, polymerase chain reactions were performed using 1X Biolase DNA Polymerase (Bioline USA, Inc., Randolph, MA) in 10 μl total volume containing 2 μl or 4 μl of stock DNA. Standard thermocycling was as follows: 94 °C for 30 s, a primer annealing temperature of 55 °C for 30 s, and an extension time of 30 s at 72 °C. As an aid to amplify some of the regions, 10% volume DMSO was added to some assays. Taqman assays were performed using a PE9700s thermal cycler with final endpoints read on a 7900HT Fast Real-Time PCR System (Applied Biosystems, Foster City, CA). These were 3 μl or 5 μl reactions using 2.2 μl or 4.5 μl of DNA diluted 1:10 from stock.

### Markers

PAGE assays were processed through polymerase chain reactions using Biolase reagents (Bioline USA, Inc.) then run out for size comparison on acrylamide gels as described previously by Lidral et al. [59]. SSCP assays were processed through polymerase chain reactions using Biolase reagents and then run out for conformation comparison on acrylamide gels as described previously by Mitchell et al. [60]. KINETIC XY was conducted as previously described by Shi et al. [61]. TaqMan was performed as previously described by Ranade et al. [62]. Allelic discrimination probe assays for SNPs were purchased from Applied Biosystems including both inventoried and noninventoried Assays on Demand as well as custom Assays By Design. Both positive and negative controls were run with all assays. Any families with apparent Mendelian errors were re-tested and then excluded from all markers if not resolved.

Markers and SNPs used in this study are shown in Table 1 and Table 2, respectively. The seven microsatellite markers on Chromosome 12 and the five on chromosome 18 were previously reported [44]. SNPs were used at other loci; three for *fibroblast growth factor 2* (basic, *FGF2*) on chromosome 4, five for *PAX6* and one for *BDNF* on chromosome 11, four for *COL2A1* on chromosome 12, and three for *COL18A1* on chromosome 18. The *PAX6* TaqMan assays listed in Table 1 were selected from Hammond et al. [46].

**Table 1:** Markers analyzed in the current study.

**Gene (chromosome)**	**Chromosomal locus**	**Variant name**	**Marker names in previous report [****44****]**
12	12q21.31	D12S2076	GATA30F04
	12q23.1	D12S1051	GATA2401
	12q23.1	D12S2081	GATA7A02
	12q23.1	D12S393	GATA15A03
	12q23.1	D12S1059	GATA47G01
	12q23.1	D12S1041	ATA24F01
	12q23.2	D12S1030	GATA6H09
18	18p11.32	D18S476	D18S476
	18p11.32	GATA178F11	GATA178F11
	18p11.31	D18S52	D18S52
	18p11.31	GATA185C06	GATA185C06
	18p11.31	GATA116D12	GATA116D12

Coverage was intended to be at least 3 markers per gene. Markers on Chromosome 12 and chromosome 18 were previously reported [44]. SNPs were used at other myopia candidate loci for *FGF2*, *PAX6*, *BDNF*, *COL2A1*, and *COL18A1*. The *PAX6* assays were selected from Hammond et al. [46]. The information presented in the "Chromosomal locus" column was obtained from the UCSC genome site.

**Table 2:** Single nucleotide polymorphisms analyzed in the current study with minor allele frequencies for parents in the sample compared to other sources.

**Gene**	**Locus**	**rs number**	**Genotyping source**	**Minor allele frequencies**
FGF2	4q27	rs1048201	AB	0.16 T
			HapMap	0.25 T
			AGI	0.13 T
			Calculated from sample parents	0.22 T
	4q27	rs1982569	HapMap	0.49 T
			Calculated from sample parents	0.44 C
	4q27	rs308447	HapMap	0.37 T
			Calculated from sample parents	0.30 T
PAX6	11p13	rs3026401	HapMap	0.17 C
			Calculated from sample parents	0.20 C
	11p13	rs662702	HapMap	0.11 T
			Calculated from sample parents	0.03 T
	11p13	rs1506	AB	0.19 T
			HapMap	0.15 T
			Calculated from sample parents	0.18 T
	11p13	rs2239789	AB	0.44 T
			HapMap	0.44 T
			Calculated from sample parents	0.46 T
	11p13	rs628224	HapMap	0.07 A
			Calculated from sample parents	0.13 A
BDNF	11p14.1	rs6265	AB	0.22 T
			HapMap	0.18 T
			AGI	0.23 T
			Calculated from sample parents	0.22 T
COL2A1	12q13.11	rs1635529	AB	0.11 T
			Calculated from sample parents	0.21 T
	12q13.11	rs1635550	AB	0.11 A
			HapMap	0.16 A
			AGI	0.21 A
			Calculated from sample parents	0.24 A
	12q13.11	rs2248990	AB	0.30 C
			Calculated from sample parents	0.49 C
	12q13.11	rs3737548	AGI	0.47 G
			Calculated from sample parents	0.26 G
COL18A1	21q22.3	rs9983675	AB	0.27 G
			Calculated from sample parents	0.37 A
	21q22.3	rs1051298	HapMap	0.42 A
			Calculated from sample parents	0.45 A
	21q22.3	rs2236479	AB	0.47 A
			HapMap	0.31 A
			Calculated from sample parents	0.19 A

Presentation of estimates from various sources allows for comparison of minor allele frequencies used in this analysis against the variability in frequency across sources. The AB, HapMap, and AGI frequencies taken from CEPH (Centre d'Etude du Polymorphisme Humain)

### Statistical analysis - genetic model for myopia

Myopia was considered to follow an autosomal dominant model with penetrance of 95% by 14 years of age [63]. Two analyses were done, one with affected status as the current diagnosis for myopia and another that took into account the age of the individual. The 51 children under the age of 14 years with no current diagnosis of myopia were indicated as having an affected status of "unknown" to indicate that they could possibly develop myopia in the future but have not yet reached their age of onset. We also applied model-free approaches using the diagnosis of myopia as the affection criterion because the genetic model for myopia is not certain. Allele frequencies were determined from the parents in the sample (Table 2). Five percent had no parents typed, ten percent had one parent typed, and 85% of the trios had both parents typed. Allele frequencies from other sources are shown for comparison. Adjustment for multiple statistical testing was made using the Bonferroni correction. Allowing for two types of statistical analyses on 28 markers, utilizing two different affection schemes (112 tests), the Bonferroni correction would indicate that a p-value of 0.0004 would be considered significant.

### Genetic analysis

For linkage analysis, we used both parametric and non-parametric approaches. We calculated model-based two-point LOD scores between myopia and each of the markers or SNPs using the Elston-Stewart algorithm [64] and employing the LINKAGE program with recent updates to speed calculations (VITESSE and FASTLINK) [65,66]. We also calculated model-free two-point analysis of the data using the Kong and Cox linear model based on IBD (identity by descent) allele sharing as implemented in MERLIN [67,68]. To detect association between the disease loci and the individual marker/SNP in the presence of linkage [69], we used the analysis package of FBAT with the additive model (v1.5.5, 2004) [70]. If weak linkage signals were detected, an empirical TDT (transmission disequilibrium test) was performed to adjust for the correlation in transmissions to multiple offspring. Haplotype analyses of specific groups of SNPs within the same gene were also completed using FBAT. Results are reported for all bi-allelic SNPs, but only significant results are reported for the microsatellite markers. FBAT allows for analysis of incomplete typed trios by using unaffected typed siblings to estimate missing parental genotypes. S.A.G.E. removes any incomplete typed trio from analysis. Reverse TDT [71] and tests of Hardy-Weinberg Equilibrium were also completed to guard against false positives. "Reverse TDT" is the assessment of transmission distortion to unaffected children rather than the usual transmission to affected children. If allelic transmission is significantly distorted from parents to both affected and unaffected children, then the transmission distortion is general and not related to the phenotype.

## Results

Table 3 lists the results for the 12 microsatellite markers and 16 SNPs genotyped for the 123 families. The results for the data, where the age of onset was considered, were no different compared to the results for the data using only the current myopia diagnosis. Therefore, only the results using the current diagnosis of myopia are reported. All "reverse TDT" analyses and non-parametric linkage analyses were not significant (results not shown).

**Table 3:** Model-free tests of association with myopia.

**Marker**	**Location**	**TDT analysis**		
		**Number of informative families**	**p-values**	
			**Bi-allelic**	**Haplotype**
**Chromosome 4**				
FGF2 rs1048201	4q27	32	0.01	
FGF2 rs1982569	4q27	42	0.13	0.59
FGF2 rs308447	4q27	25	0.83	
**Chromosome 11**				
PAX6 rs3026401	11p13	22	0.62	
PAX6 rs662702	11p13	8	NR	
PAX6 rs1506	11p13	4	NR	
PAX6 rs2239789	11p13	56	0.89	0.79
PAX6 rs628224	11p13	24	0.22	
BDNF rs6265	11p14.1	29	0.67	
**Chromosome 12**				
COL2A1 rs1635529	12q13.11	44	0.00007	
COL2A1 rs1635550	12q13.11	34	0.19	0.78
COL2A1 rs2248990	12q13.11	65	0.54	
COL2A1 rs3737548	12q13.11	58	0.13	
D12S2076 (GATA30F04)	12q21.31	37	NS	
D12S1051 (GATA2401)	12q23.1	32	NS	
D12S2081 (GATA7A02)	12q23.1	42-46	NS	
D12S393 (GATA15A03)	12q23.1	10-60	NS	
D12S1059 (GATA47G01)	12q23.1	17	NS	
D12S1041 (ATA24F01)	12q23.1	10-59	NS	
D12S1030 (GATA6H09)	12q23.2	32-54	NS	
**Chromosome 18**				
D18S476	18p11.32	12-31	NS	
GATA178F11	18p11.32	10-16	NS	
D18S52	18p11.32	11-37	NS	
GATA185C06	18p11.32	14-38	NS	
D18S967 (GATA116D12)	18p11.32	20-61	NS	
**Chromosome 21**				
COL18A1 rs9983675	21q22.3	58	0.84	
COL18A1 rs1051298	21q22.3	63	0.85	0.77
COL18A1 rs2236479	21q22.3	30	0.24	

The significant finding from the analysis is an association between myopia and *COL2A1* (rs1635529). The p value for *FGF2* (rs1048201) is not significant after correction for multiple comparisons. The significant p value is marked in red. In the table, NR indicates the p-value was not reported due to an insufficient number of informative families. Also, NS indicates the p-value is not significant for any allele with a sufficient number of informative families.

On chromosome 12, strong evidence of association between myopia and the common allele of the *COL2A1* SNP rs1635529 was found (p=0.00007, with 44 informative families) along with weak evidence for linkage (parametric LOD of 1.11). Assuming the presence of linkage with the empirical calculation of the test statistic, repeating the TDT analysis resulted in a p-value of 0.0008, which is above the Bonferroni level of 0.0004 but still of interest. The reverse TDT analysis was insignificant (p=0.50, 26 informative families). In analyzing only the 101 Caucasian families, this *COL2A1* SNP rs1635529 was significant at the p=0.0005 level with 33 informative families, again above the Bonferroni level of 0.0004 but still of interest. Reverse TDT on this Caucasian sample was also insignificant (p=0.37, 23 informative families). The haplotype TDT analysis for the *COL2A1* SNP group was not significant (p=0.78). Other TDT results following correction for multiple comparisons were not significant for microsatellite markers D12S2076 (GATA30F04), D12S1051 (GATA2401), and D12S1059 (GATA47G01) in the biallelic analysis.

None of the three SNPs within the *FGF2* group showed significant findings from the different analysis methods after correction for multiple comparisons. There was some evidence of association in the TDT analysis between myopia and over-transmission of the common allele of the *FGF2* SNP rs1048201 (p=0.01). In the two-point dominant model linkage analysis, the *FGF2* SNP rs308447 had a LOD score of 0.95. The haplotype TDT analysis for this *FGF2* SNP group was not significant (p=0.59) and the non-parametric linkage was not significant for any SNP in this group. Within the *PAX6* group, no SNP demonstrated statistical significance in any of the analyses. Lack of informative families for two of the *PAX6* SNPs made the association between myopia and these SNPs unknown. Elsewhere on chromosome 11, weak evidence for linkage was found with the *BDNF* SNP rs6265 (LOD=1.19).

On chromosome 18, all analyses for the microsatellite markers were performed on a sufficient number of informative families yet, there were no significant findings (p greater than or equal to 0.16 for biallelic analyses and p greater than or equal to 0.46 for multi-allelic analyses). On chromosome 21, all of the collagen *COL18A1* SNPs had sufficient number of informative families yet, none had significant results from any of the analyses.

## Discussion

We used a large sample size and a range of genetic models and test strategies to search for genetic causes of myopia using candidate genes and loci. Myopia is a complex trait where analyses are complicated by the likely involvement of multiple genes, gene-gene interactive effects, and the need for large sample sizes to detect effects. A candidate gene approach as opposed to a genome-wide approach is obviously limited by gene and SNP choice. However, SNP coverage could not be complete given the availability of assays at the time of the study, the associated costs, and the limited amount of genetic material available from our buccal swabs. Coverage was intended to be at a density of at least three markers per gene. The recent publication of a genome-wide, dense-haplotype map makes it practical to consider genome-wide association approaches [72]. One such application in an ocular disease, age-related macular degeneration, has already been successful [73]. Nonetheless, these approaches are labor and resource intensive, so using a subset of well-chosen candidates affords practical possibilities for gene identification. The candidates in this study were used in previous investigations of single gene disorders predisposed to myopia (*COL2A1* and Stickler Syndrome [54], *COL18A1* and Knobloch Syndrome [53]). Others were chosen for their potential biological relevance to myopia (*FGF2* [47,74], *BDNF* [52], and *PAX6* [46]). Other candidates were loci in proximity to regions that have previously been suggested through linkage analysis to be associated with various forms of myopia [34-36].

The most significant association for myopia seen in the current study was for *COL2A1* with a p-value of 0.00007. This is somewhat surprising since, typically, myopia is regarded as a condition of excessive axial, scleral growth, yet, human sclera is predominantly comprised of type I collagen with little to no evidence for the presence of type II collagen [75]. Type II collagen, however, is a primary constituent of vitreous [76], although vitreous has never been considered a major factor in determining refractive error. It is possible that the association for *COL2A1* is due to a shared linkage disequilibrium pattern with a causative variant in a nearby gene, but the two known adjacent genes, *vitamin D receptor* (*VDR*) and *SUMO1/sentrin specific peptidase 1* (*SENP1*) are not obvious candidates. However, there is some evidence that variations in type II collagen might affect the development of the eye. Comparisons between normal and transgenic mice with deletions or mutations for type II collagen show that expression of type II collagen mRNA is widespread throughout the eye during development with the transgenic mice showing reduced filament density in vitreous, anterior displacement of iris and lens, shallow anterior chamber, and disorganized structure to cornea and lens [77]. Common forms of myopia share ocular abnormalities with Stickler syndrome such as myopic refractive error, cataract, glaucoma, and retinal detachment [2-8] but have none of the facial, auditory, or joint abnormalities seen in some forms of Stickler syndrome [78]. These shared ocular traits raise the interesting question of whether the ocular sequelae of ordinary myopia have any overlap in etiology with Stickler syndrome or whether the glaucoma and detachment seen in Stickler syndrome are in part consequences of the increased axial length associated with common myopia. The myopia of Stickler syndrome does differ from common myopia by being congenital, severe, and often associated with a membranous appearance to the vitreous or with a chorioretinal degeneration [78,79]. Several studies have excluded linkage between high, pathological forms of myopia and candidate gene regions for type II collagen [34,37,39,41]. The association seen in the current study may be due to either the majority of our subjects having less than pathological amounts of myopia or the fact that we used the TDT rather than a linkage approach. For complex human traits, association approaches such as the TDT may be more sensitive than linkage approaches [80].

The failure to find additional, positive results with the association-based approach does not exclude any particular gene from involvement considering the sample size and the presence of an unknown level of heterogeneity. Linkage can find signals for etiologic genes when there is allelic heterogeneity, but the current study had only modest power to detect linkage. Ethnic diversity in the sample may be a weakness because it may add to heterogeneity. However, we believe there is value in evaluating candidates that are represented across ethnic backgrounds. Subject numbers for individual ethnic groups seem insufficient for analyses of association on a group-by-group basis. However it is worth noting that the allele frequencies calculated from all parents in this ethnically diverse sample are similar to those from the predominantly Caucasian data taken from CEPH (Table 2). Another possible limitation is misclassification due to the measurement of the right eye only in probands. However, the prevalence of anisometropia is low, on the order of less than 4% [81-83]. Parents and siblings might also be misclassified based on survey responses. The survey used has a reported sensitivity of 0.76 and a specificity of 0.74 [57].

Unlike Hammond et al. [46], we were unable to demonstrate any significant linkage of myopia to *PAX6*. Using the same five SNPs used by Hammond et al. [46], we were unable to show any association with myopia. The two studies differed in sample composition in that the current study concentrated on myopia while Hammond et al. had a sample with a wider spectrum of refractive errors. However, their linkage signal was maintained, whether they examined myopes or hyperopes as separate groups [46]. Clearly the analysis of this region needs to be expanded to include nearby genes or regulatory loci.

Expression of FGF-2 is altered in animal deprivation studies but has not been evaluated previously in human studies. In the tree shrew, the experimental induction of myopia did not result in differences in the level of scleral FGF-2 compared to control eyes, but there was upregulation of FGF receptor mRNA in experimental myopia [74]. The specific mechanism by which FGF-2 might influence eye growth in experimental myopia has not been defined, but it may exert influence through regulation of scleral fibroblast proliferation or by stimulation of proteases during scleral reformation. We did not see evidence of the involvement of *FGF2* in myopia in our sample.

Three markers on chromosome 12 (D12S2076, D12S1051, and D12S1059) continued to show no significant associations with myopia in this larger sample, consistent with a previous analysis of a subset of the samples used in the current study [44]. A previous report using a large sample of 78 families with a careful analysis of study power and similar definitions of myopia to the current study also did not find any strong evidence of linkage of myopia to the 12q21-23 or 18p11.31 regions [45].

In summary, the primary findings from the current study suggest involvement in common forms of myopia by *COL2A1*. There was no significant evidence for involvement by *FGF2*, *BDNF*, or *COL18A1.* No significant associations were found for markers on chromosomes 12 and 18 (previously linked with high myopia) and the more common forms of myopia in the present study. Consistent with a report by others, we were unable to demonstrate an association between *PAX6* and myopia.
